# Social encounter networks: collective properties and disease transmission

**DOI:** 10.1098/rsif.2012.0357

**Published:** 2012-06-20

**Authors:** Leon Danon, Thomas A. House, Jonathan M. Read, Matt J. Keeling

**Affiliations:** 1 Mathematics Institute, University of Warwick, Gibbet Hill Road, Coventry CV4 7AL, UK; 2 School of Life Sciences, University of Warwick, Gibbet Hill Road, Coventry CV4 7AL, UK; 3 Department of Epidemiology and Population Health, Institute of Infection and Global Health, University of Liverpool, Leahurst Campus, Neston CH64 7TE, UK

**Keywords:** social contact, epidemic, infectious disease, power law, survey

## Abstract

A fundamental challenge of modern infectious disease epidemiology is to quantify the networks of social and physical contacts through which transmission can occur. Understanding the collective properties of these interactions is critical for both accurate prediction of the spread of infection and determining optimal control measures. However, even the basic properties of such networks are poorly quantified, forcing predictions to be made based on strong assumptions concerning network structure. Here, we report on the results of a large-scale survey of social encounters mainly conducted in Great Britain. First, we characterize the distribution of contacts, which possesses a lognormal body and a power-law tail with an exponent of −2.45; we provide a plausible mechanistic model that captures this form. Analysis of the high level of local clustering of contacts reveals additional structure within the network, implying that social contacts are degree assortative. Finally, we describe the epidemiological implications of this local network structure: these contradict the usual predictions from networks with heavy-tailed degree distributions and contain public-health messages about control. Our findings help us to determine the types of realistic network structure that should be assumed in future population level studies of infection transmission, leading to better interpretations of epidemiological data and more appropriate policy decisions.

## 1. Introduction

The study of the spread of directly transmitted infections is intimately linked to the science of networks. For a given disease, potential routes of transmission between individuals form edges of a network that, when combined, can connect entire populations [1–3]. The pervasive and highly connected nature of these networks is illustrated by the rapid progression around the world of both SARS and H1N1 pandemic influenza [4,5]. For many infectious diseases, potential transmission opportunities occur whenever two people are in close social contact; therefore the transmission network is a suitably scaled version of the social contact network. A full dynamic quantification of this social contact network for an entire population would be a powerful tool, allowing an accurate prediction of real-time disease spread. Such a goal is, however, practically unachievable; yet even a partial understanding of the general structure of a transmission network can yield a wealth of valuable insights. Statistical characterizations of networks such as scale-free, small-world or locally clustered are all known to be associated with particular patterns of disease spread and alternative mechanisms of optimal control [6–8]. Quantifying potential transmission routes and the complex structure of the associated network is therefore a major challenge for infectious disease epidemiology, with benefits for public health.

For respiratory and close contact infections (such as influenza, measles or meningitis), previous studies have attempted to quantify appropriate social encounters through questionnaires [9,10]. The use of such data has highlighted the potential of social contact networks to better describe the routes of transmission [2,11,12] and provide insights into the effects of shifting social interaction patterns on disease incidence [13]. However, until now, large-scale questionnaire-based surveys have not sought to capture either extreme behaviour, or local network structure. Electronic proximity sensors have the capability to provide such information [14,15], but are limited to recording interactions between individuals participating in the study, restricting their ability to identify individuals with many contacts and providing only a partial picture of the local network.

Social interactions are also pivotal in spreading ideas and influencing individual behaviour. This has been observed in several health-related contexts: both smoking and obesity, among others, are strongly correlated with the behaviour of close contacts [16,17]; awareness of epidemic threat propagating through the social network can reduce the speed and extent of the disease [18]; the local structure of the social network has additional influence on the adoption of healthy behaviour, with messages reinforced through tight knit, clustered networks amplifying the spread of behaviour [19]. Our understanding of social dynamics would therefore benefit from a more detailed quantitative understanding of social network structures.

**Figure 1.**
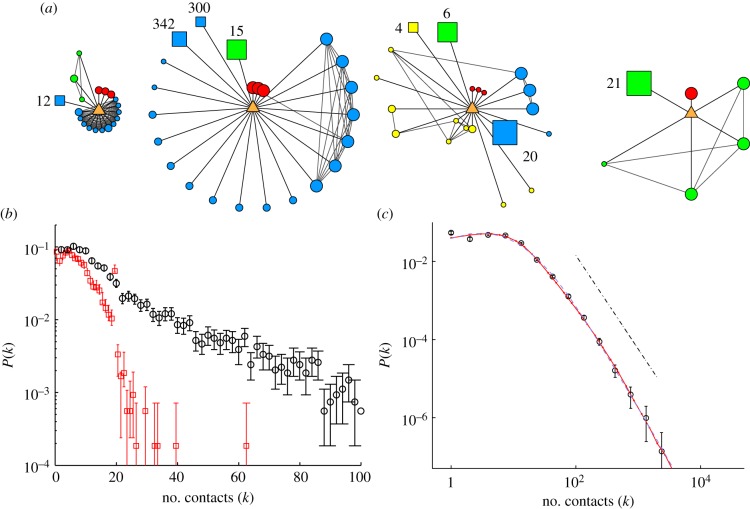
Capturing individual contact heterogeneity. (*a*) Examples of ego-centric networks collected by the survey. From left to right: school pupil, female aged 12 years; flight attendant, female 22; fire fighter, male 44; retired, male 62. The participant (ego) is the orange central triangle; circles represent individual contacts, and squares represent groups of contacts (size of group indicated). Colours represent social settings of encounters (red, home; blue, work/school; yellow, travel; green, other). Larger symbol sizes represent longer contact durations, while a closer proximity to the ego indicates the contact is more frequently encountered. (*b*) The distribution of contacts (node degree) from the survey with no group information included (red squares) and with groups included (black circles). (*c*) The distribution of the number of contacts (node degree) from the survey (open circles), compared with our model of daily contacts (red line; see electronic supplementary material), and a guide to the eye line following a power-law decay with exponent of −2.45. Confidence intervals of the distribution are determined by bootstrapping (open circles, with groups; red line with dots, model; blue dashed line, dPlN fit; dashed-dotted line, slope =−2.4).

To address these gaps in our knowledge, we conducted a cross-sectional survey of the population within Great Britain (GB), with the aim of characterizing all close contacts. Surveys were sent to 140 000 randomly selected households in GB with information from additional participants being collected through an online version of the questionnaire that was open to anyone regardless of their nationality or location (see electronic supplementary material for more details). Participants were requested to volunteer their basic demographic data as well as all their social contacts for a single day (cf. [9,10]). Participants were asked to note down each face-to-face conversation (within 3 m/10 feet) during the course of a day. This included encounters with physical contact and explicitly excluded virtual interactions. Each contact could either refer to a single individual, or a group of individuals all met at the same time (e.g. a dinner party of eight people) or to a number of individuals that were all met separately but in a similar context (e.g. serving several different customers). In addition, information was sought on the intimacy, context, location, duration and frequency of each encounter. Local structure of the contact network surrounding each participant was captured by asking which contacts were likely to have encountered each other on the day in question or during the previous 7 days. The postal survey was complemented with an online version, which allowed us to access a wider cross section of the population and to continue collecting information. Figure 1*a* shows four examples of local networks captured by our surveys. An example of the postal questionnaire is shown in the electronic supplementary material, figure S1, and the online version can be found at www.contactsurvey.org. By October 2010, we had collected information on 5388 individuals or egos (5027 of whom were located in GB) and 145 329 secondary contacts, which forms the basis of this study. Further details on the survey respondents are reported in the electronic supplementary material.

## 2. Data, notation and processing

To enable us to be more explicit in our calculation of various quantities of interest, we first define some notation to quantify the answers of each respondent. For a given respondent *i* in our survey, we can enumerate all of their contacts *c* = 1, …, *k*_*i*_, where *k*_*i*_ is the degree of respondent *i*. In the questionnaire, we allowed individuals to report ‘groups’ of individuals that were met in similar contexts; to simplify the later formulations such groups are inflated into constituent individuals, so that if a respondent reports contact with a group of five people it is replaced by five individual contacts. We further define that the first *K*_*i*_ contacts are associated with individual contacts, while the remaining *k*_*i*_ – *K*_*i*_ contacts are derived from groups. Each contact (*c* of an individual *i*) has an associated context *C*_c_^*i*^ (which could be home, work/school, travel and other), distance from home *D*_c_^*i*^ where the contact occurred, frequency of meeting *F*_c_^*i*^, and duration *T*_c_^*i*^. In addition, our survey collects information on transitive connections between contacts, asking whether two contacts had met in the past week; *W*_*c,d*_^*i*^ is defined to be one if such a connection exists between contacts *c* and *d* of individual *i*, or zero otherwise.

Two modifications are required to these raw data to allow a simple analysis of the patterns and trends. First, the total duration of contact, *T*_c_^*i*^, is reported as lying within one of four discrete intervals (less than 10 min, between 10 and 30 min, between 30 min and an hour, or over 1 h). To allow a single-point value to be calculated for many quantities of interest, these intervals (*T*_c_^*i*^) for each contact are translated into random variables, *t*_c_^*i*^, chosen from a stretched exponential distribution that fits the aggregate data (see electronic supplementary material for details). All confidence intervals in this paper incorporate the uncertainty arising from picking these random variables.

The second modification concerns the time-scale differences between contacts, which are recorded for a single day, and connections between contacts (*W*_*c,d*_^*i*^), which are based on one week. To consider these measures on an equal footing, we inflate the contacts to form a week-long network by including multiple copies of contacts (see electronic supplementary material). This process produces a new set of contacts (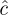 of which there are 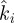), transitive links (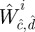) and contact durations (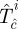). This inflation assumes that all days are similar, such that for a contact that is encountered for the first time that day, we would expect seven such contacts to occur in a week, whereas a contact that is encountered most days will be present for most of the week. Any daily snap-shot of this inflated network returns the original sample. This inflation makes a minimal assumption about transitive links and does not introduce links between contacts met on different days; therefore, calculated levels of clustering are likely to be consistent underestimates of actual values. Again, all results given in the paper include the confidence intervals associated with this inflation process.

## 3. Degree distribution

The most fundamental characterization of a person's immediate social network is the total number of social contacts or the individual's degree; this is the key to assessing both their risk of infection and their potential for onward transmission [20]. Hence, population-level distributions of this degree are crucial to understanding the dynamics of epidemics, with theoretical studies showing that heavy-tailed distributions lead to qualitatively different disease dynamics.

In particular, scale-free degree distributions that generate an infinite variance in the infinite population limit can give rise to disease dynamics with no epidemic threshold but where targeting control at a small proportion of highly connected individuals is very effective at reducing transmission [7,8].

Such distributions have been observed in electronic proxies for social contacts [14,21], have emerged in synthetic populations [22], and have been inferred for sexual contacts [23] (although these are difficult to confirm statistically due to the lack of samples of sufficient size [24]), but have not previously been directly recorded for social interactions.

Previous surveys of social contacts have generally limited the number of contacts that are recorded in detail, simply due to logistical constraints [9,10], which places an artificial bound on the contact distribution. By allowing respondents the flexibility to report groups of individuals as well as contacts with single individuals, our questionnaire has alleviated the burden of reporting large numbers of contacts. Figure 1*b* shows the reported distribution of individual-only contact (ignoring groups and showing the distribution of *K*_*i*_) compared with the full distribution when group data are included (distribution of *k*_*i*_). There is a clear censoring issue for individual-only contacts, with a notable peak at *K* = 20 the maximum number of individual contacts that could be reported on the paper questionnaire.

In addition, the two measurements, *k*_*i*_ and *K*_*i*_, yield qualitatively different forms for the distribution of contacts. When groups are ignored, the degree distribution is well modelled by a negative binomial distribution (with *r* = 3.0 and *p* = 0.26, see electronic supplementary material, SI4A) as observed in previous studies [10]. However, when groups are taken into account, the distribution is found to possess a lognormal body with a power-law tail with an exponent of −2.45 ± 0.2 for numbers of contacts *K* above 28. Such a distribution is poorly fitted by standard forms (see electronic supplementary material, figure S6B), and we must adopt alternative approaches to capture this distribution. The first approach is statistical and involves fitting a more complex functional form to the data. The double Pareto lognormal distribution (*dPlN*, blue dashed line in figure 1*c*) allows for both a lognormal body and the power-law tail, and has recently been proposed to model heavy-tailed distributions associated with income growth [25] and degree distributions for mobile phone networks [26]. The fitting procedure and best-fit of the dPlN distribution are shown in the electronic supplementary material.

The second approach is to derive a plausible mechanistic model (red line, figure 1*c*) that captures the distribution. We assume that individuals make new contacts during the day at a heterogeneous rate (*ρ*, chosen from a lognormal distribution), with the contact chosen through preferential attachment [27] such that highly connected individuals are more likely to be contacted. In particular, we assume that an individual who currently has *n* connections is contacted by other people at a rate proportional to a function *f*(*n*). We take *f*(*n*) = *n* + *α* , and so when *α* = 0 we have the preferential attachment model of Barabási & Albert [27]; but when *α* becomes large connections form at random (and for a fixed *ρ* value this generates an Erdös–Rényi network). The probability density for an individual with contact rate *ρ* having *n* contacts at time *t* is given by

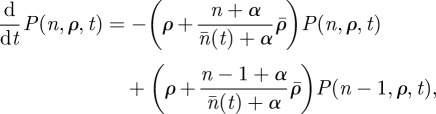

where the average number of contacts across the population 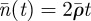. The distribution of contacts that is of interest is the distribution at the end of the day (*t* = 1 as we are interested in all contacts made in a given day), i.e. the proportion of individuals with *n* links is 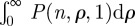. This model captures the shape of the distribution well and provides an intuitive, plausible mechanism for the creation of real social encounters during a single day. We find that assuming that the distribution of contact rates (*ρ*) is lognormal produced a good fit to the distribution of contacts (*k*_*i*_) when 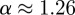 and 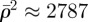. Hence, the best-fit distribution of contact rates is highly overdispersed 
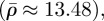
 and the model requires a relatively strong degree of preferential attachment (the electronic supplementary material provides more details).

## 4. Local structure

We now focus on a second novel aspect of our survey, the transitive (triangle forming) links between contacts, that determines the clustering within the local egocentric networks. The simplest measure would be to calculate, for each individual, the proportion of pairs of contacts that are connected by a transitive link:

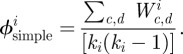

Although we report such values, there is a potential bias in this calculation: the contacts are from a single day, whereas transitive links are reported for an entire week. To equalize these measures, we inflate the contacts to form a one-week network by including multiple copies of contact types (see the electronic supplementary material). Using the associated weekly measures, we can calculate an unweighted measure of clustering that captures the topology of the local network:

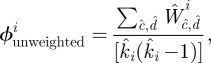

and a weighted measure of clustering that accounts for the time spent with each contact:

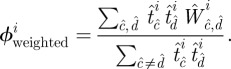

In general, clustering is high with means of around 0.38, 0.07 and 0.11 for simple, unweighted and weighted measures, respectively.

However, more important than the mean values is the relationship between clustering and degree (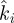). For networks where links between individuals are formed by a homogeneous process that connects free half-links (for example, in the configuration model [28] or the recently proposed generalization [29]), theoretical considerations predict that the unweighted (topological) clustering must scale like 1/(*k* – 1). The intuition behind this argument is relatively straightforward: if a highly connected individual has high clustering, then each of its contacts must be connected to many of its other contacts; this means that each contact must also have high degree, which breaks the random-connection assumption implicit in the network formation. Our clustering measures show exactly this behaviour. While the level of clustering declines with increasing degree, the rate of decline is far slower than the theoretically expected 1/(*k* – 1) decline. Therefore, the network of social connections must be degree-assortative, with high-degree nodes more likely to connect to other high-degree nodes than expected at random. Degree assortativity is epidemiologically very important, inflating the basic reproductive ratio due to greater-than-expected transmission within the high-degree, ‘core group’ of the population [20]. (Some estimation of the strength of this assortativity is given in the electronic supplementary material.)

We now wish to understand the underlying mechanism that drives this high degree of clustering within the network. In the literature, two commonly used mechanisms for generating clustered networks are membership of different cliques [30] or spatially localized contact formation [31]. We investigate these by considering the transitive matrix (often named *confusion matrix*); this measures the proportion of times a transitive link is present between two contacts that are either made in specified contexts or at specified distances from home. For two contexts *A* and *B*, the transitive matrix is defined as

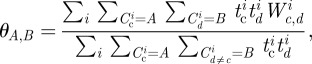

where the sum is performed over all *c* and *d* where the information on transitive links is available. Here, we are using a weighted measure based on the time spent with each contact, which we feel is more biologically motivated; a similar formulation holds for the transitive matrix based on distance from home (see the electronic supplementary material for more details).

**Figure 2.**
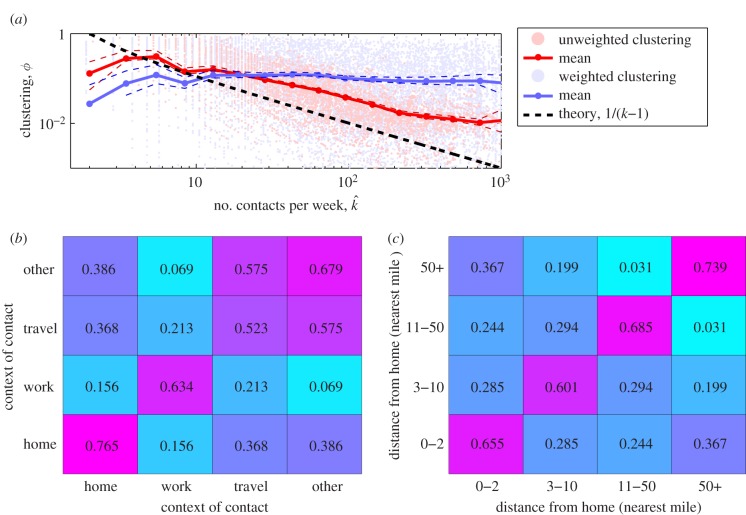
Local clustering of contacts. (*a*) Relationship between number of contacts reported, 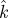, and the clustering between those contacts (red, unweighted; blue, weighted); confidence intervals are determined by bootstrapping. (*b*,*c*) Transitive matrices showing the degree of clustering stratified by social context and distance from home; the values are the proportion of transitive links between contacts in different setting compared with the theoretical maximum.

Our results do not conform to the predictions of either clique or spatial models. Although transitivity is highest between contacts in similar social contexts, significant transitivity between contexts is also observed, which would not be present if contacts are generated through clique membership alone. We note that work-related contacts form the most cohesive group that is isolated from other social contexts (figure 2*b*). In addition, transitivity does not decay with distance as expected if a purely spatial process is involved (figure 2*c*); instead we find the highest cohesion occurs between contacts made over 50 miles from home, suggesting that when people travel a significant distance, they are more likely to meet as a highly interconnected group. These findings show that the social processes that generate clustering are complex, driven by the movement of individuals in space and the overlapping contexts within which interactions occur.

## 5. Epidemiological implications

**Figure 3.**
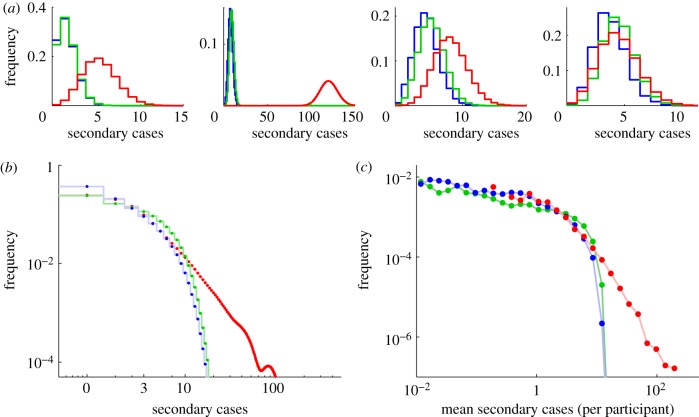
Epidemiological implications of local network structure: three network models are compared: a simple (unweighted, unclustered) network (shown in red), a weighted network accounting for duration of contact (green) and a clustered weighted static network accounting for the full structure around participant (blue). (*a*) Distributions of the number of secondary cases (*R*_*i*_) for the four examples in figure 1*a*. (*b*) Distribution of secondary cases across the entire sample of participants (*R*_*i*_). (*c*) Distribution of expected number of secondary cases per participant (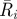). We have modelled a short-lived, rapidly transmitted infection, with a latent period of 3 days, an infectious period of 3 days (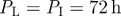), and a transmission rate, *τ*, of 0.1 h^− 1^ across a network connection.

The collection of data on social contacts was motivated by a desire to better understand the spread of directly transmitted infections. Although we have shown that the social encounter network must be scale-free in the tail, highly clustered and therefore degree-assortative, there are insufficient data to parametrize and build a full social network without making some strong assumptions about how the recorded ego-networks interconnect. We therefore assess the epidemiological implications of individual heterogeneity and the network structure surrounding each participant by calculating individual level reproductive ratios (figure 3; cf. [32]). Assuming that a randomly chosen contact is infected (and the remainder are susceptible), we can calculate both the distribution (*R*_*i*_, figure 3*a*,*b*) and the expected number 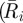, figure 3*c*) of secondary cases generated by the participant, if they become infected.

We consider three forms of heterogeneous network: (i) an unweighted unclustered network, where each contact is assumed to be present for the mean duration (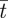, approx. 39 min d^−1^) and transitive links are removed (figure 3, red); (ii) a weighted, unclustered network, where contact duration is specified for each contact in the survey (blue); and (iii) a weighted clustered network (green). The number of secondary cases can be calculated via direct simulation using the following basic methodology: we form the network around the respondent and choose a random contact to be infected; we monitor the ensuing epidemic and record the number of secondary cases caused by the respondent if they become infected. Throughout, we assume fixed-duration latent and infection periods (*P*_L_ and *P*_I_, respectively) and a fixed transmission rate *τ* across a contact for the duration of infection. This method of repeated simulation is currently the only viable approach when the network is clustered; however when clustering is ignored (that is we remove the links between contacts in the local network), then analytic results can be obtained (see the electronic supplementary material, §5).

For the weighted clustered network, it is necessary to simulate the epidemic process and to assign a weight for the transitive link between contacts. As a relatively minimal assumption about the role of clustering, we set the duration of the transitive link between two contacts (if it exists) as the minimum of the duration associated with the two contacts, as such information was too complex to collect in our survey.

There are two measures that characterize the transmission of disease: the expected number of secondary cases that an individual will generate, 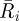, which captures the between individual variation; and the distribution of secondary cases, *R*_*i*_, which is closer to observations taken of an epidemic and incorporates the stochastic nature of transmission. Differences between the network formulations are best captured by the expected number of secondary cases, 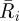. In the unweighted, unclustered network, the expected number of secondary cases 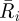 follows a power law with mean 4.62 and variance 167.75; such high variance is expected with a power-law tail in accordance with the power-law network paradigm. For the weighted, un-clustered network, the tail of the distribution is curtailed such that the mean of the expected number of secondary cases is substantially reduced to 2.97 with the variance reduced by an order of magnitude to 8.59. Including the transitive links (the most complete network formation) does not radically change the shape of the distribution, but does lead to many more situations where no subsequent secondary cases are generated and the mean of the expected number of cases is further reduced to 1.96 with variance 5.91. In this final network formulation, the distribution of secondary cases is well modelled by a negative binomial form (NB(*r*,*p*) where *r* = 0.86 and *p* = 0.31), which agrees with estimates made during epidemics [32]. These results highlight the role of contact duration and clustering in predicting the spread of infection and in dampening extremes of behaviour.

## 6. Conclusions

Here we have reported on results from a postal and online questionnaire. As with all such surveys, several issues emerge that could bias our results. The most obvious of which is that we have to trust that respondents provide reliable and accurate information; however given that all information is provided voluntarily with no incentive, we believe that there are no reasons why respondents should seek to mislead. Some biases inevitably exist, such as a propensity for older and more educated individuals to complete the survey, but our qualitative findings are robust to such biases.

Results from our ego-centric contact data provide strong insights on the types of structures that are present in social contact networks: a moderate to high level of clustering, degree assortativity and power-law topology. The reported contact degree of respondents to our survey was highly over-dispersed with mean and variance of 26.97 and 5194, respectively, giving a coefficient of variation of 2.67; in addition, this degree distribution exhibits a power-law tail with a small proportion of the population having a very high number of contacts. If we were to extrapolate the tail of the distribution to infinite population sizes, the variance would diverge (in practice, the tail of the distribution and therefore the variance are always bounded). Such highly heterogeneous degree distributions are generally associated with high basic reproductive ratios, relatively small final epidemic sizes and an inability to control infection by random vaccination [32].

Even though a power-law assumption holds for individuals with high numbers of contacts, the epidemiological consequences are also related to the duration of contact, and physical constraints mean that having many contacts limits the duration that can be spent in close proximity with each. The action of contact duration will generally dampen or eliminate the impact of the associated heavy tail [33], and the distribution of the mean number of cases can be well fitted by a negative binomial distribution, thus bridging the gap between power laws often measured or calculated for degree distributions [14,21,22] and negative binomial distributions often reported for secondary cases [32]. These results indicate that models ignoring contact duration will systematically overestimate both the rates of spread and the importance of high-frequency, short-duration contacts [31,34].

Despite the dampening effect of contact duration, the implications of heterogeneities in contact structure are still strong: our epidemiological simulations predict that around 90 per cent of infections originate from the 50 per cent of the population with the highest levels of transmission, while the heterogeneity in contact duration means that 90 per cent of transmission is to the 40 per cent of contacts with the longest total durations. These findings suggest that contact tracing, which is often implemented during the early stages of novel epidemics, could be substantially targeted with certain types of contact being low priority.

Local clustering patterns within networks are known to have important consequences for public health, including contact-tracing-based interventions due to the multiple routes via which infected people can be traced [35,36], vaccine uptake patterns [37], the optimal deployment and critical level of vaccination [38], and more generally the rate of spread of infection, ideas and behaviour [19]. Here we measured clustering in three ways; the most naive measure simply considered whether two contacts were thought to have met each other during the past 7 days. While this time scale for contacts meeting was epidemiologically motivated, the mixture of a single day for reported contacts against 7 days for transitive links would produce an over-estimate of clustering compared with standard network measures. To overcome this issue, we inflated reported contacts to 7 days (assuming a similar pattern of social interaction on each day), allowing us to estimate clustering for the 7-day network. Even though our approach is likely to produce an underestimate of true clustering, all estimates are seen to be above the theoretical threshold for configuration networks. This means that the social network must be degree-assortative, implying that high-degree nodes are more likely to connect to other high-degree nodes.

In the light of all these findings, policy decisions based on the predictions of random transmission models or simple network assumptions should be re-evaluated. The effects of network structure are known to change the relationships between early epidemic growth rates, final epidemic sizes and peak number of cases [39] and hence an accurate characterization of the network is vital if early data are to inform public health policy. In addition, the heterogeneities detected in our survey highlight the potential for targeted control and refined contact tracing during an epidemic. It remains to be seen how these results translate to other populations, where cultural, demographic and social influences may be different from GB, or where extremes of rural and urban living generate a wider spectrum of population density.
